# Assessment of Hyoid Bone Position and its Correlation With Airway Dimensions in Different Sagittal Malocclusions Using Cone Beam Computed Tomography

**DOI:** 10.1155/ijod/6702204

**Published:** 2025-05-26

**Authors:** Priya Singh, Supriya Nambiar, Asavari Desai, Nidhin Philip, Ravikiran Ongole

**Affiliations:** ^1^Department of Orthodontics and Dentofacial Orthopaedics, Manipal College of Dental Sciences Mangalore, Manipal Academy of Higher Education, Manipal, Karnataka 576104, India; ^2^Department of Oral Medicine and Radiology, Manipal College of Dental Sciences Mangalore, Manipal Academy of Higher Education, Manipal, Karnataka 576104, India

## Abstract

**Objectives:** To evaluate the changes in the position of the hyoid bone in different skeletal malocclusions and correlate it with pharyngeal airway dimension using cone beam computed tomography (CBCT).

**Materials and methods:** Ninety healthy adult subjects between 18 and 25 years with normo divergent facial pattern (FMA range 21° and 28°) from the postgraduate orthodontic clinic of the Institution. The subjects were divided into three groups consisting of 30 patients each, on the basis of the ANB angle. Each group was subdivided into two according to gender to evaluate if any sexual dimorphism exists in airway dimensions and hyoid bone position.

**Results:** Oropharyngeal volume was maximum in Class I group (15.18) and least in Class II group (12.06), which was statistically significant with a *p* value of 0.015. Horizontal distance from highest point of the hyoid bone and true vertical in Class II group had the highest value of 21.51 and Class I had the least value of 17.31, which was statistically significant with *p* value of 0.027. Vertical measurement between the hyoid bone and the posterior nasal spine (PNS) in Class III subjects was around 57.01, and Class I subjects had the least value of 51.20. Nasopharyngeal volume was higher in the male group with a *t* value of −2.798 and was statistically significant.

**Conclusion:** The hyoid bone is posteriorly positioned in Class II skeletal pattern. Oropharyngeal volume was least in Class II skeletal base individuals. In males, pharyngeal volume was larger than females, and the hyoid bone was placed inferiorly compared to females.

## 1. Introduction

Respiration through the upper airway is an essential physiological process, and any alteration in this process at the time of facial growth can influence the normal facial development. The hyoid bone has connections to the mandible, pharynx, and cranium through muscles and ligaments, and most importantly, is the only bone that does not have any direct bony articulation; thereby making it necessary for vital functions such as talking, chewing, swallowing, and airway patency [1]. Thurow postulated that the function of the geniohyoid muscle was to adjust the antero–posterior position of the hyoid and maintain the airway patency throughout the various craniofacial complex movements [2]. Position of the hyoid bone at the level of the genial tubercle increases the muscular efficiency in forward movement of the tongue and maintaining the airway [3]. Various options available for assessment of the pharyngeal space are cephalometric radiography, acoustic reflection, nasopharyngoscopy, conventional and electron beam computed tomography, three-dimensional computed tomography, MRI/volumetric MRI, and optical coherence tomography are the few techniques widely employed for imaging of upper pharyngeal airway.

Upper pharyngeal airway space has been most widely evaluated by cephalometric radiographs, which can be obtained in two views: postero–anterior (PA) and lateral cephalometric view. Jose et al. [4] observed that a positive correlation existed between the oropharyngeal airway and horizontal distance from the hyoid bone to retrognathion in Class I skeletal pattern. Lateral cephalometric view can lead to superimposition of the bilateral structures of the facial skeleton, provides a two-dimensional visualization, which is not accurate as it involves the complex structures and has other severe limitations, such as distortions, low reproducibility due to difficulties in landmark identification, and differences in magnification. Due to these limitations, two-dimensional analysis ends up examining the hard and soft tissues around the airway, rather than investigating just the airway space. Hence, the reproducibility of airway dimensions and tongue and hyoid position on lateral cephalometric radiographs is proven to be unreliable [5, 6].

The advent of cone beam computed tomography (CBCT) has shifted the paradigm of maxillofacial imaging from two-dimensional to three-dimensional approaches of data acquisition and image reconstruction, thereby creating a revolution in dental diagnosis. Images generated from the reconstructed views have already proven to be useful in diagnosis and treatment planning of impacted teeth, surgical orthodontic care and temporomandibular joint (TMJ) pathology, planning of dental implants, pathological lesion identification, supernumerary teeth location, mixed dentition planning, and airway analysis. CBCT studies have been done to correlate hyoid bone position and airway space postinterventions like functional appliance or orthognathic surgery. The present study was done to evaluate the positional changes of hyoid bone in different skeletal malocclusions and to assess the correlation between the pharyngeal airway dimension and the hyoid bone position using CBCT.

## 2. Materials and Methods

This retrospective study was approved by the Institutional Review Board (ref no: 16111), and pretreatment records of 90 healthy adult subjects between 18 and 25 years with normo divergent facial pattern (FMA range of 21°–28°) were selected. Subjects with any gross dental abnormalities, oral habit, or any history of previous orthodontic treatment were excluded. Subjects with a history of diseases affecting the pharyngeal structures were also excluded. The power of the study was set at 80% with 95% confidence interval, according to which a minimum of 84 subjects would the required power of the study. Based on the sample calculation, a sample of 90 patients was decided for the study. The selected records were categorized into three groups consisting of 30 patients each, on the basis of the ANB angle.

I. Class I skeletal group: ANB angle between 0° and 3°.
II. Class II skeletal group: ANB angle more than or equal to 4°.
III. Class III skeletal group: ANB angle less than 0.

Each group was subdivided into two according to gender to evaluate if any sexual dimorphism exists in airway dimensions and hyoid bone position.

**Figure 1:**
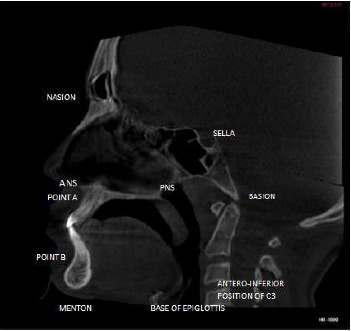
Landmarks seen in the sagittal section.

The three-dimensional scans were obtained using a CBCT scanner (PlanmecaProMax 3D Mid; PlanmecaOy, Helsinki, Finland) at 90 kV, 5.6 mA, and a field of view of 200 mm × 170 mm [7]. The subjects were scanned for 6 s while standing upright with the head in a natural position in centric occlusion (Figure 1). The CBCT data stored in digital images and communication in medicine (DICOM) format was exported to the Planmeca Romexis software for the analysis. Only pretreatment CBCT scans taken as part of the routine preorthodontic investigations were used from the archive of the Department of Orthodontics and Dentofacial Orthopedics.

**Figure 2:**
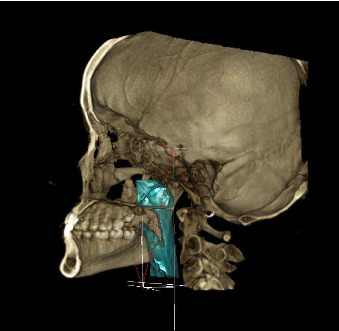
3D reconstructed view of airway created using airway measurement tool.

The three-dimensional (3D) reconstruction of the patient's head was oriented so that the FH plane was parallel to the axial plane and the midsagittal plane was oriented to the subject's midline. The patient's midsagittal plane is defined as a vertical plane passing through both the anterior nasal spine (ANS) and the posterior midpoint of the spine (centrum) (Figure 2). Thirteen landmarks were labeled in the midsagittal view [8]. Landmarks that were used is as depicted in (Table 1, Figure 1). The hyoid bone position was evaluated using eight linear parameters:

I. C3–Me;
II. H–Me;
III. C3–H;
IV. H–X;
V. H–EB;
VI. H–Y;
VII. H–PNS;
VIII. H– (C3–Me);

and two angular parameters:

i. H–S–Ba.
ii. H–N–S.

Oropharyngeal and nasopharyngeal volume was measured using the following landmarks [5] as depicted in Table 1. Description of the three-dimensional pharyngeal airway parameters is given in Table 2. The landmarks were identified and located on the sagittal section, and analysis was done. For airway analysis, the area of interest was selected and matched in all three views: sagittal, coronal, and axial (Figure 2). The threshold limit was set at 500 for all the subjects.

## 3. Results

**Figure 3:**
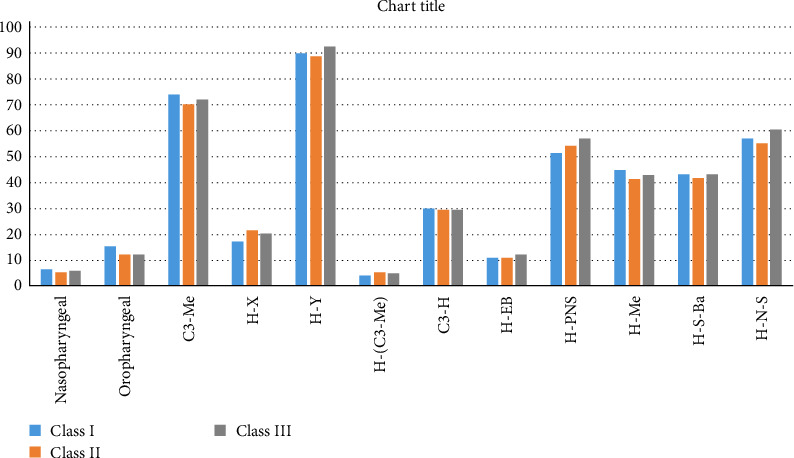
Intergroup comparison of airway and hyoid bone parameters in Class I, II & III malocclusion.

Oropharyngeal volume was highest in Class I and least in Class II groups, which was statistically significant. Post Hoc Tukey test comparing Class I and Class II groups and Class I and Class III groups showed a mean difference of 3.12 and 2.96, respectively, and the values were statistically significant (Table 3, Figure 3).

**Figure 4:**
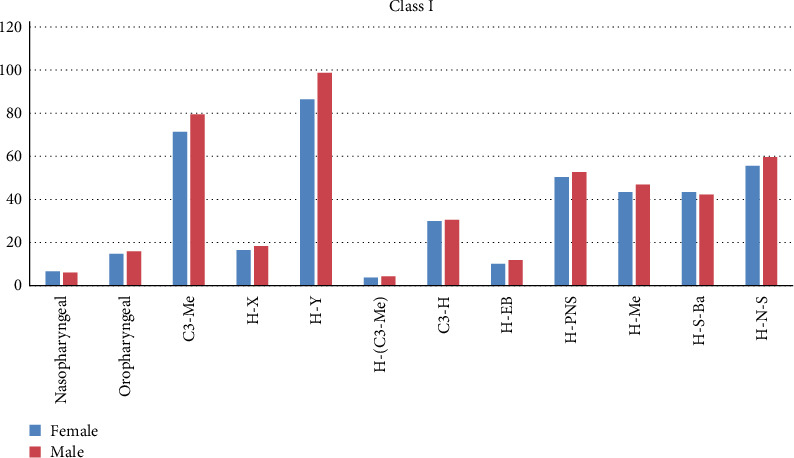
Gender based comparison of airway and hyoid bone parameters in Class I malocclusion subjects.

Horizontal distance from the highest point of the hyoid bone and true vertical passing through sella (H–X) was highest in Class II and least in Class I group, which was statistically significant. Post Hoc Tukey test comparing Class I and Class II groups is shown in Table 4 and Figure 4.

Vertical measurement between the hyoid bone and posterior nasal spine (PNS) was found to be highest in Class III subjects and least in Class I subjects, which was statistically significant. Post Hoc Tukey test comparing Class I and Class III groups is shown in Table 4.

The linear measurement between the hyoid bone and menton was found to be highest in Class I group and least in Class II group. Post Hoc Tukey test comparing Class I and Class II groups showed a mean difference of 3.28*⁣*^*∗*^ and was statistically significant with a *p* value of 0.012.

**Figure 5:**
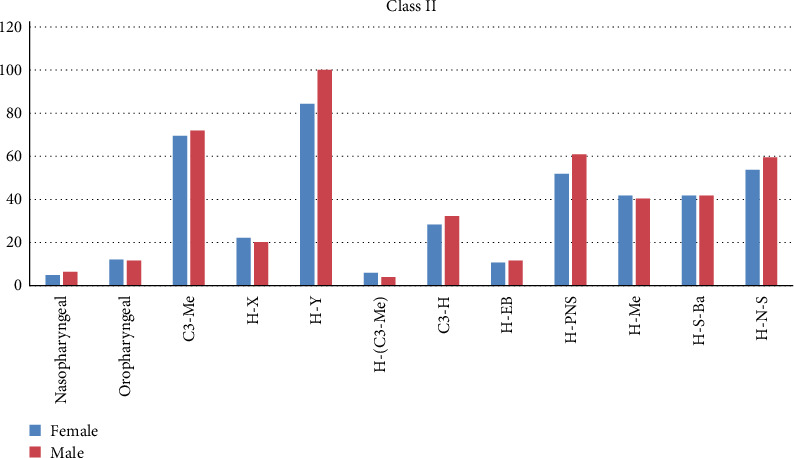
Gender-based comparison of airway and hyoid bone parameters in Class II malocclusion subjects.

Anterior cranial base angle from hyoid-nasion-sella (H-N-S) was highest in Class III group and least in Class II that was statistically significant. Post Hoc Tukey test comparing Class II and Class III groups showed a mean difference of −5.31 and was statistically significant with a *p* value of 0.04 (Table 5, Figure 5).

In Class I subjects, nasopharyngeal volume showed a strong positive correlation with H-(C3-Me) and H-S-Ba. Nasopharyngeal volume and anterior cranial base angle, H-N-S showed a positive correlation whereas with C3-Me, it showed a good negative correlation. Oropharyngeal volume showed a strong positive correlation with H-(C3-Me), C3-Me, and C3-H, and strong negative correlation with H-PNS (Table 5).

**Figure 6:**
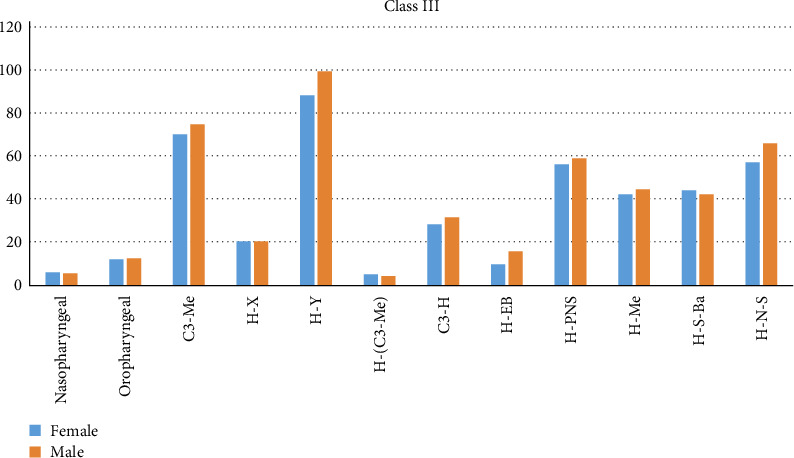
Gender based comparison of airway and hyoid parameters in Class III subjects.

Linear measurements, C3-Me, H-Y, and H-EB were higher in the male group among Class I subjects (Table 6, Figure 6). Among Class II subjects, nasopharyngeal volume was higher in males and were statistically significant. Linear parameters H-Y, C3-Me, H-PNS, and angular parameter H-N-S were higher in males and was statistically significant (Table 6, Chart II). In Class III subjects, H-Y, H-EB, and H-N-S were higher in males and were statistically significant (Table 6, Figure 6).

## 4. Discussion

The hyoid bone is the only bone in the human body that does not have any bony articulation and has muscular and ligamentous attachment to structures, such as cranium, mandible, and pharynx, and plays an important role in maintenance of airway, speech, swallowing, and upright postural position of head. Orthodontic and orthognathic interventions can disrupt the balance between airway space and the hyoid bone position, and hence any information pertaining to influence of different sagittal positions of jaw bases on these structures can improve the diagnosis and treatment [1].

This was a retrospective study done using the images obtained from CBCT scan done under standardized conditions and retrieved from the archives of the Department of Orthodontics and Dentofacial Orthopedics. For better reliability and accuracy three-dimensional CBCT scans were used as per the findings of Lagravere et al. [9] and Baumgaertel et al. [10]. Due to the retrospective nature of the study, direct assessment of nasorespiratory pattern of each subject was not possible. Hence, the selection was done based on case history and clinical examination, where any subject with signs and symptoms of pharyngeal pathology was excluded. The power of the study was set at 80% with 95% confidence interval, according to which minimum of 84 subjects would be the required power of study. This was in accordance with the study done by Jiang et al. [8]. The samples were collected from adult patients aged between 18 and 25 years of age to rule out any developmental changes in the position of the hyoid bone and pharyngeal volume as reported by Sheng et al. [11]. King et al. [12] reported that the dimensions of the pharynx continued to grow rapidly until 13 years of age, and then there is a minimal growth until adulthood.

De Freitas et al. [13] and Opdebeeck et al. [14] reported that subjects with vertical growth pattern have narrow upper airway. Hence, in this study, only normodivergent subjects were included (FMA between 21° and 28°) to eliminate any effect on nasopharyngeal airway caused by different growth patterns of mandible. Sexual dimorphism was also evaluated between the hyoid bone parameters and pharyngeal airway volume in the subjects, especially nasopharyngeal airway as has been seen in the study by Tofangchiha et al. [15]. Jose et al. [4] found that the horizontal distance between the hyoid bone and retrognathion point was higher in males. Nasopharyngeal volume was highest in Class I subject and least in Class II subjects. This is contrary to the result of the study done by Hong et al. [16], where they found that nasopharyngeal airway volume was largest in patients with skeletal Class III malocclusion. Class III malocclusion can also be due to retrognathic maxilla, where nasopharyngeal volume will be reduced. Oropharyngeal volume was found to be lowest in Class II subjects. This can be explained by Balter's philosophy, according to which in Class II malocclusions, the tongue is positioned backward, thereby disturbing the cervical region. This could possibly lead to reduction in the oropharyngeal volume in such patients. The distance of hyoid bone to the true vertical passing through sella was found to be highest in class II subjects and least in Class I subjects, and the comparison between Class I and Class II subjects was found to be statistically significant. This can be attributed to the changes in craniocervical angulation in Class II subjects. Hosseinzadeh et al. [17] reported a significant correlation between cervical column posture angles and parameters, such as ANB and Wits in Class II patients. Nobili and Adverse et al. [18] pointed to the body posture at various dysnathia and concluded that people with distocclusion keep their head slightly forward as opposed to people with mesiocclusion, who hold their head back, thus indirectly changing the position of hyoid bone. Hyoid bone to PNS was found to be highest in Class III subjects and least in Class II subjects. This can be explained by the anterior positioning of hyoid bone in Class III subjects and posterior position of hyoid bone in Class II subjects and this was in agreement with the result of the study done by Mortazavi et al. [19]. The distance between the hyoid bone and menton was highest in Class I subjects and least in Class II subjects, and comparison between Class I and Class II subjects was found to be statistically significant suggesting that retrognathic mandible places the hyoid bone posteriorly due to its muscular attachment [4]. Mortazavi et al. [19] reported that hyoid bone is positioned more posteriorly in Class II skeletal patterns. Angular parameters were found to be highest in Class III subjects and lowest in Class II subjects, indicating that hyoid bone is positioned anteriorly and superiorly in Class III subjects and vice versa in Class II subjects.

Nasopharyngeal volume and H-(C3-Me), anterior and posterior cranial base were strongly correlated in Class I subjects, indicating that the nasopharyngeal volume has a positive correlation with vertical position of the hyoid bone. This may explain why any alteration in position of the hyoid bone by mandibular movement can cause changes to upper airway space. Oropharyngeal volume was found to be positively correlated with H-(C3-Me) and C3-H. Trenouth et al. [20] reported that oropharyngeal size was positively correlated to the length of mandible (gon-men), distance between C3 and the hyoid bone, and cranial base angle. Oropharyngeal volume was negatively correlated with H-PNS. A study reported by Harlabakis et al. [21] showed a strong inclination of hyoid bone in relation to the palatal plane. In Class II subjects, oropharyngeal volume was positively correlated with C3-Me and C3-H. Delji et al. [22] found a positive correlation between the hyoid bone and cervical vertebrae, and also found it to be associated to mandibular plane. Whereas oropharyngeal volume was found to be negatively correlated with H-N-S, which indicated that the hyoid bone was placed posteriorly in Class II subjects. This is in agreement with the result of the study done by Mortazavi et al. [19].

In Class I subjects, both nasopharyngeal and oropharyngeal volumes were found to be greater in males than in females. Li et al. [23] reported a longer length of palatopharynx and oropharynx and larger oropharyngeal volume in boys over 13 years of age. Sheng et al. [11] showed that the values C3-H, H-C3-Me, H-PNS, and H-EB were larger in boys than in girls. Our study showed similar results, where H-EB and H-Y were found to be greater in males than in females. Nasopharyngeal volume was higher in males, whereas the oropharyngeal volume was found to be higher in females in Class II subjects. Mortazavi et al. [19] reported that the hyoid bone is positioned superiorly and posteriorly in females than males, and its location differs in different skeletal patterns. It is placed more posteriorly in skeletal Class II patterns and inferiorly and anteriorly in Class I pattern. In Class II subjects, H-Y, C3-H, H-PNS, and H-N-S were found to be larger in males. This was in agreement to the result of the study done by Jiang et al. [24]. In Class III subjects, H-Y, H-EB, and H-N-S were found to be larger in males and were statistically significant.

## 5. Conclusion

Based on the observations of this study, it can be concluded that hyoid bone is posteriorly positioned in Class II skeletal pattern and anteriorly in Class I skeletal pattern with respect to the true vertical. Oropharyngeal volume was the least in Class II skeletal base individuals compared to Class I and Class III skeletal bases. In males, pharyngeal volume was larger than females and the hyoid bone was placed inferiorly compared to females. In Class I skeletal pattern, both nasopharyngeal and oropharyngeal volumes were directly proportional to the vertical distance of the hyoid bone from the plane connecting the third cervical veterbra and mandible. Nasopharyngeal volume was directly proportional to anterior and posterior cranial base angles. Oropharyngeal volume was directly proportional to linear distance of hyoid bone to third cervical vertebra, and vertical distance of hyoid bone to PNS. In Class II skeletal base individuals, oropharyngeal volume was directly proportional to the linear distance of hyoid bone to third cervical vertebra, hyoid bone to menton, and hyoid bone to anterior cranial base angle. In Class III skeletal pattern, nasopharyngeal volume was inversely proportional to vertical distance of the hyoid bone to PNS.

## Figures and Tables

**Table 1:** Description of landmarks.

Sella (*S*)	Geometric center of the pituitary fossa located by visual inspection
Nasion (*N*)	Located on the most anterior aspect of the frontonasal suture
Basion (Ba)	Median point of the anterior margin of the foramen magnum
Point A	Deepest point in the midline between the anterior nasal spine and alveolar crest between two central incisor
Point B	Deepest point in the midline between the alveolar crest of mandible and the mental process
Menton (Me)	Most inferior midline point on the mandibular symphysis
ANS	Anterior tip of nasal spine
PNS	Intersection between continuation of anterior wall of pterygopalatine fossa and floor of the nose
C3	The most antero–inferior point on the corpus of the third cervical vertebra
Frankfort horizontal plane (FH plane)	Plane connecting the lowest point of the orbit (orbitale) and the superior point of the external auditory meatus (porion)
Pterygomaxillary point (PTM point)	Intersection of inferior border of the foramen rotundum with the posterior wall of the pterygomaxillary fissure
Posterior nasal spine (PNS)	The intersection of a continuation of anterior wall of the pterygopalatine fossa and floor of the nose, marking the distal limit of the maxilla

**Table 2:** Description of the three-dimensional pharyngeal airway parameters.

Variable	Description
Nasopharyngeal airway	Anterior border is a line connecting pterygomaxillary point (PTM) and posterior nasal spine (PNS). Inferior border is a plane parallel to the Frankfort horizontal plane through the PNS. Posterior border is the posterior wall of the pharynx. The volume between these landmarks was calculated.

Oropharyngeal airway	Superior border is a plane passing parallel to the Frankfort horizontal (FH) plane through the PNS (the inferior border of the nasopharyngeal airway.) Inferior border is a plane passing through antero inferior point of third cervical vertebra parallel to FH plane. The volume between the landmarks was calculated from the software.

**Table 3:** Intergroup comparison of airway volume and C3-Me in Class I, II, and III subjects using one way ANOVA and Post Hoc Tukey test.

	Groups	*N*	Mean	Std. deviation	Welch statistics (*⁣*^*∗*^)/F (ANOVA)	*p* Value
Nasopharyngeal volume	Class I	30	6.4762	2.12	2.079	0.131
	Class II	30	5.385967	1.54		
	Class III	30	6.010567	2.46		
	Total	90	5.957578	2.10		

Oropharyngeal volume	Class I	30	15.1858	5.11	4.395	**0.015**
	Class II	30	12.06287	3.82		
	Class III	30	12.22127	4.76		
	Total	90	13.15664	4.77		

C3-Me	Class I	30	73.966	8.18	2.409	0.096
	Class II	30	69.97033	4.68		
	Class III	30	71.828	7.77		
	Total	90	71.92144	7.16		

Dependent variable		(I) group	(J) group	Mean difference (I–J)	Std. error	*p* Value
Nasopharyngeal volume		Class I	Class II	1.090233	0.536576	0.111
			Class III	0.465633	0.536576	0.662
		Class II	Class III	−0.6246	0.536576	0.478

Oropharyngeal volume		Class I	Class II	3.1229333*⁣*^*∗*^	1.186588	**0.027**
			Class III	2.9645333*⁣*^*∗*^	1.186588	**0.038**
		Class II	Class III	−0.1584	1.186588	0.99

C3-Me		Class I	Class II	3.995667	1.822005	0.078
			Class III	2.138	1.822005	0.472
		Class II	Class III	−1.85767	1.822005	0.567

*⁣* ^*∗*^indicates statistical significance with the *p*-value in bold.

**Table 4:** Intergroup comparison of hyoid bone position in Class I, II and III subjects using one way ANOVA and Posthoc Tukey test.

	Groups	*N*	Mean	Std. deviation	Welch statistics (*⁣*^*∗*^)/F (ANOVA)	*p* Value
H-X	Class I	30	17.31	4.68	3.874*⁣*^*∗*^	**0.027**
	Class II	30	21.52	8.44		
	Class III	30	20.35	6.47		
	Total	90	19.72	6.87		

H-Y	Class I	30	90.20	7.77	1.427	0.246
	Class II	30	88.56	9.36		
	Class III	30	92.30	8.53		
	Total	90	90.35	8.62		

H-(C3-Me)	Class I	30	4.10	2.78	1.106	0.336
	Class II	30	5.24	2.79		
	Class III	30	4.90	3.48		
	Total	90	4.75	3.04		

C3-H	Class I	30	30.15	5.10	0.258	0.773
	Class II	30	29.47	3.52		
	Class III	30	29.31	5.51		
	Total	90	29.64	4.75		

H-EB	Class I	30	10.87	2.50	1.06	0.351
	Class II	30	10.95	3.48		
	Class III	30	12.08	4.54		
	Total	90	11.30	3.61		

H-PNS	Class I	30	51.20	8.98	3.15	**0.048**
	Class II	30	54.22	6.62		
	Class III	30	57.01	10.79		
	Total	90	54.14	9.18		

H-Me	Class I	30	44.61	4.43	4.33	0.016
	Class II	30	41.33	4.21		
	Class III	30	42.96	4.31		
	Total	90	42.97	4.48		

H-S-Ba	Class I	30	43.22	7.08	0.653	0.523
	Class II	30	41.71	4.77		
	Class III	30	43.26	5.92		
	Total	90	42.73	5.98		

H-N-S	Class I	30	56.94	6.64	3.147	**0.048**
	Class II	30	55.07	5.60		
	Class III	30	60.39	11.50		
	Total	90	57.46	8.52		

Dependent variable		(I) group	(J) group	Mean difference (I–J)	Std. error	*p* Value
H-X		Class I	Class II	−4.2060*⁣*^*∗*^	1.734	**0.045**
			Class III	−3.045	1.734	0.191
		Class II	Class III	1.1613	1.734	0.782

H-Y		Class I	Class II	1.64	2.216	0.74
			Class III	−2.095	2.216	0.613
		Class II	Class III	−3.735	2.216	0.217

H-(C3-Me)		Class I	Class II	−1.136	0.785	0.322
			Class III	−0.802	0.785	0.566
		Class II	Class III	0.334	0.785	0.905

C3-H		Class I	Class II	0.681	1.237	0.847
			Class III	0.836	1.237	0.778
		Class II	Class III	0.155	1.237	0.991

H-EB		Class I	Class II	−0.0847	0.932	0.995
			Class III	−1.215	0.932	0.397
		Class II	Class III	−1.1303	0.932	0.449

H-PNS		Class I	Class II	−3.0223	2.315	0.396
			Class III	−5.8093*⁣*^*∗*^	2.315	**0.037**
		Class II	Class III	−2.787	2.315	0.454

H-Me		Class I	Class II	3.2860*⁣*^*∗*^	1.117	**0.012**
			Class III	1.651	1.117	0.306
		Class II	Class III	−1.635	1.117	0.313

H-S-Ba		Class I	Class II	1.513	1.551	0.594
			Class III	−0.0437	1.551	1
		Class II	Class III	−1.557	1.551	0.577

H-N-S		Class I	Class II	1.8693	2.150	0.661
			Class III	−3.4477	2.150	0.25
		Class II	Class III	−5.3170*⁣*^*∗*^	2.150	**0.04**

*⁣* ^*∗*^indicates statistical significance with the *p*-value in bold.

**Table 5:** Pearsons correlation for assessment of the correlation of the hyoid bone parameter and the airway volume in Class I subjects.

Parameters	*N*	Class I		ClassII		Class III	
		Corr (*r*)	*p* Value	Corr (*r*)	*p* Value	Corr (*r*)	*p* Value
Nasopharynx volume & C3-Me	30	−0.197	0.297	0.043	0.82	−0.469	**0.009**
nasopharynx volume & H-X	30	−0.077	0.684	−0.034	0.857	0.11	0.563
nasopharynx volume & H-Y	30	−0.165	0.385	0.262	0.162	0.11	0.564
nasopharynx volume & H-(C3-Me)	30	0.431	**0.017**	−0.056	0.771	−0.094	0.622
nasopharynx volume & C3-H	30	0.279	0.136	0.291	0.119	0.006	0.975
nasopharynx volume & H-EB	30	0.136	0.474	−0.049	0.796	−0.115	0.546
nasopharynx volume & H-PNS	30	−0.333	0.072	0.169	0.372	0.131	0.49
Nasopharynx volume & H-Me	30	0.028	0.883	−0.161	0.394	−0.132	0.487
Nasopharynx volume & H-S-Ba	30	0.52	**0.003**	−0.021	0.911	0.186	0.326
Nasopharynx volume & H-N-S	30	0.381	**0.038**	0.119	0.532	−0.23	0.222
Oropharynx volume & C3-Me	30	0.215	0.254	0.455	**0.011**	−0.226	0.229
Oropharynx volume & H-X	30	0.359	0.051	−0.009	0.962	−0.031	0.87
Oropharynx volume & H-Y	30	−0.033	0.862	−0.162	0.392	0.285	0.127
Oropharynx volume & H-(C3-Me)	30	0.468	**0.009**	0.215	0.254	−0.097	0.61
Oropharynx volume & C3-H	30	0.412	**0.024**	0.468	**0.009**	−0.007	0.971
Oropharynx volume & H-EB	30	0.21	0.265	−0.166	0.382	0.158	0.406
Oropharynx volume & H-PNS	30	−0.511	**0.004**	−0.125	0.512	0.257	0.17
oropharynx volume & H-Me	30	0.273	0.145	0.392	**0.032**	0.196	0.3
Oropharynx volume & H-S-Ba	30	0.309	0.096	−0.177	0.35	−0.039	0.84
Oropharynx volume & H-N-S	30	0.141	0.458	−0.367	**0.046**	−0.167	0.376

*Note:* Bold indicates statistical significance.

**Table 6:** Independent *t*-test showing intergroup comparison between males and females in Class I subjects.

	G	*N*	Class I S			Class II					Class III				
			Mean	SD	*p*	G	*N*	Mean	SD	*p*	G	*N*	Mean	SD	*p*
Nasopharynx	F	21	6.60	2.13	0.629	F	22	4.95	1.33	**0.009**	F	19	6.12	2.48	0.741
	M	9	6.18	2.15		M	8	6.56	1.55	—	M	11	5.80	2.54	—

Oropharynx	F	21	14.7	4.49	0.507	F	22	12.16	3.58	0.81	F	19	11.91	4.57	0.652
	M	9	16.15	6.54		M	8	11.77	4.65	—	M	11	12.74	5.25	—

C3- Me	F	21	71.51	7.94	**0.01**	F	22	69.26	4.61	0.177	F	19	70.11	4.34	0.114
	M	9	79.68	5.71		M	8	71.90	4.60	—	M	11	74.78	11.22	—

H-X	F	21	16.7	5.01	0.284	F	22	21.93	7.93	0.657	F	19	20.46	5.65	0.909
	M	9	18.73	3.69		M	8	20.35	10.24	—	M	11	20.17	7.98	—

H-Y	F	21	86.57	5.11	**<0.001**	F	22	84.39	5.98	**<0.001**	F	19	88.21	6.45	**<0.001**
	M	9	98.69	6.18		M	8	100.05	7.06	—	M	11	99.36	7.04	—

H-(C3-Me)	F	21	4.00	2.52	0.762	F	22	5.73	2.72	0.111	F	19	5.24	3.97	0.492
	M	9	4.34	3.47		M	8	3.88	2.69	—	M	11	4.32	2.47	—

C3-H	F	21	29.84	3.47	0.721	F	22	28.47	3.10	**0.008**	F	19	28.14	2.97	0.128
	M	9	30.86	7.97		M	8	32.20	3.28	—	M	11	31.34	8.06	—

H-EB	F	21	10.28	2.30	**0.047**	F	22	10.65	3.27	0.449	F	19	9.89	2.19	**<0.001**
	M	9	12.24	2.54		M	8	11.77	4.12	—	M	11	15.86	5.12	—

H-PNS	F	21	50.43	4.81	0.48	F	22	51.82	5.48	**<0.001**	F	19	55.98	13.03	0.502
	M	9	53.01	15.13		M	8	60.82	4.90	—	M	11	58.79	5.18	—

H-Me	F	21	43.69	4.11	0.081	F	22	41.64	4.35	0.508	F	19	42.10	3.57	0.154
	M	9	46.77	4.65		M	8	40.46	3.94	—	M	11	44.45	5.21	—

H-S-Ba	F	21	43.56	7.23	0.699	F	22	41.73	4.53	0.96	F	19	43.85	4.14	0.486
	M	9	42.44	7.09		M	8	41.63	5.73	—	M	11	42.25	8.32	—

H-N-S	F	21	55.71	6.12	0.125	F	22	53.50	4.76	**0.009**	F	19	57.26	3.90	**0.049**
	M	9	59.8	7.30		M	22	4.95	1.33	—	M	11	65.78	17.49	—

*Note:* Bold indicates statistical significance.

## Data Availability

The data are available with the authors and will be shared only on request.
